# Sensitivity to Auditory Spectral Width in the Fetus and Infant – An fMEG Study

**DOI:** 10.3389/fnhum.2013.00917

**Published:** 2013-12-31

**Authors:** Jana Muenssinger, Tamara Matuz, Franziska Schleger, Rossitza Draganova, Magdalene Weiss, Isabelle Kiefer-Schmidt, Annette Wacker-Gussmann, Rathinaswamy B. Govindan, Curtis L. Lowery, Hari Eswaran, Hubert Preissl

**Affiliations:** ^1^fMEG Center, University of Tuebingen, Tuebingen, Germany; ^2^Institute for Medical Psychology and Behavioral Neurobiology, University of Tuebingen, Tuebingen, Germany; ^3^Department of Phoniatrics and Pediatric Audiology, Clinic of Otorhinolaryngology, Head and Neck Surgery, St. Elisabeth-Hospital Bochum, Bochum, Germany; ^4^Department of Obstetrics and Gynecology, University Hospital Tuebingen, Tuebingen, Germany; ^5^Department of Neonatology, University Children’s Hospital Tuebingen, Tuebingen, Germany; ^6^Division of Fetal and Transitional Medicine, Children’s Hospital, Washington, DC, USA; ^7^Department of Obstetrics and Gynecology, University of Arkansas for Medical Sciences, Little Rock, AR, USA

**Keywords:** WN, spectral width, auditory change detection, magnetoencephalography, auditory evoked responses

## Abstract

Auditory change detection is crucial for the development of the auditory system and a prerequisite for language development. In neonates, stimuli with broad spectral width like white noise (WN) elicit the highest response compared to pure tone and combined tone stimuli. In the current study we addressed for the first time the question how fetuses react to “WN” stimulation. Twenty-five fetuses (*M*_age_ = 34.59 weeks GA, SD ± 2.35) and 28 healthy neonates and infants (*M*_age_ = 37.18 days, SD ± 15.52) were tested with the first paradigm, wherein 500 Hz tones, 750 Hz tones, and WN segments were randomly presented and auditory evoked responses (AERs) were measured using fetal magnetoencephalography (fMEG). In the second paradigm, 12 fetuses (*M*_age_ = 25.7 weeks GA, SD ± 2.4) and 6 healthy neonates (*M*_age_ = 23 days and SD ± 6.2) were presented with two auditory oddball conditions: condition 1 consisted of attenuated WN as standard and 500 Hz tones and WN as deviants. In condition 2, standard 500 Hz tones were intermixed with WN and attenuated WN. AERs to volume change and change in spectral width were evaluated. In both paradigms, significantly higher AER amplitudes to WN than to pure tones replicated prior findings in neonates and infants. In fetuses, no significant differences were found between the auditory evoked response amplitudes of WN segments and pure tones (both paradigms). A trend toward significance was reached when comparing the auditory evoked response amplitudes elicited by attenuated WN with those elicited by WN (loudness change, second paradigm). As expected, we observed high sensibility to spectral width in newborns and infants. However, in the group of fetuses, no sensibility to spectral width was observed. This negative finding may be caused by different attenuation levels of the maternal tissue for different frequency components.

## Introduction

Auditory change detection is an important prerequisite for a functional auditory system as well as for the development of language perception and can serve as an indicator for healthy cognitive functioning and development. It has been repeatedly shown that humans possess already at birth the capacity to process acoustic regularities and react to violations of such regularities, meaning that they are able to detect and discriminate different patterns of sound (Carral et al., 2005). Prior studies showed atypical auditory evoked responses (AERs) to auditory changes in pre-maturely born infants (Fellman et al., 2004) as well as in infants with genetic risk for dyslexia (Leppänen et al., 1999). However, the neurophysiological mechanisms underlying early sound perception and discrimination within both, typical and untypical development, are still not fully understood.

Most studies investigating change detection used oddball paradigms and evaluated AERs elicited by individual stimuli and mismatch negativity (MMN) responses determined by the difference between AERs elicited by standard and deviant stimuli. AERs are neurophysiologic indices of sensory functioning and their different components reflect basic cognitive functions. MMN (Näätänen, 2001) – a negative component which in adults peaks at around 150 ms after change onset – is considered as an indicator of automatic change detection reflecting discriminatory capacity. Assessing healthy infants, several studies showed that they were able to distinguish between pure tones of different frequencies (Kushnerenko et al., 2002; Huotilainen et al., 2003; Draganova et al., 2005, 2007). Kushnerenko et al. (2002) studied the development of change detection in neonates over the first 12 months and did not find statistically significant differences between the responses at different ages on the group level. They also showed that changes between pure tones and novel sounds (clicks, chirps, vowels, syllables) at the age of 2 years elicited significantly higher AER responses to novel sounds compared to pure tones. This effect might be explained by the “novelty” of the stimulus itself or could be caused by the change in spectral width between stimuli, because the novel sounds were composed of different frequencies. To test both hypotheses, Kushnerenko et al. (2007) investigated responses to pure tones, novel sounds, and white noise (WN) segments. WN segments were chosen to match the novel sounds in the broad frequency range but not in the novelty. Results revealed the highest responses for WN segments. This indicates that – unlike adults – neonates are highly sensitive to the effective amount of stimulation, with broader widths eliciting higher AERs. This finding is also supported by behavioral data showing that infants are more sensitive to the amount of stimulation than to the modality of stimulation (Lewkowicz and Turkewitz, 1980; Turkewitz et al., 1983).

However, as shown in several studies using fetal magnetoencephalography, auditory change detection occurs already in the fetal stage (Draganova et al., 2005, 2007; Huotilainen et al., 2005). MMN responses were observed as early as 28 weeks of gestation. In these studies, pure tones with a frequency of 500 Hz were used as standard stimuli, which were intermixed with rarely presented deviants with a frequency of 750 Hz. However, to our best knowledge, fetal responses to more complex stimuli have not been investigated so far. One potential difficulty in using more complex stimuli in fetuses is the sound attenuation caused by maternal tissue. In general, it is known that high frequencies are more attenuated than lower frequencies [for review see Hepper and Shahidullah (1994)]. When using complex stimuli composed of multiple frequencies, the broad difference in attenuation levels between frequencies might result in the fact that fetuses perceive only parts of the whole frequency spectrum. This involves a decrement in the perceived differences between pure tones and complex stimuli by the fetus.

However, studies investigating fetal learning support the assumption that a broad spectrum of sounds reach the fetus *in utero*. Starting at the 29th week of gestational age, music as well as speech sounds were repeatedly presented to fetuses (Partanen et al., 2013a,b). Electroencephalographic (EEG) measurements directly after birth as well as 4 months after birth (Partanen et al., 2013a) showed enhanced AERs to the “learned” stimulus. AERs were significantly higher than in a control group of age-matched infants who were not presented with specific auditory stimulation before birth. This indicates that fetuses are able to perceive and learn a wide range of auditory stimuli *in utero*.

The aim of the first part of the current study (performed in Tuebingen) was to use a simplified paradigm to replicate the findings of Kushnerenko et al. (2007) in neonates and infants and to evaluate if the human fetal brain is able to process and react to differences in spectral width. Similar to newborns and infants, it was expected that fetuses will be sensitive to stimulus energy and will show increased AER amplitudes in reaction to the high spectral width of WN segments compared to those elicited by pure tones. In the second part of the study (performed in Little Rock), a within group oddball paradigm including both changes in volume and spectral width was used to further explore fetal cortical responses to different auditory changes. Higher AERs were expected for changes in frequency than for changes in volume.

## Materials and Methods

### Data acquisition

Data was recorded using SQUID Array for Reproductive Assessment (SARA) systems installed in two different locations, one at the Department of Obstetrics and Gynecology, University of Arkansas for Medical Sciences, AR, USA and a second one at the fMEG Center, University of Tuebingen. Both systems were built by VSM, Medical Technology Ltd., Canada. To avoid magnetic influences from the environment, the systems are installed in shielded rooms (Vakuumschmelze, Germany). The system in Little Rock contains 151 primary sensors and the system in Tuebingen 156 primary sensors. The primary sensors measure the biomagnetic signals from the physiological sources. Little Rock data were recorded with a sampling rate of 312.5 Hz and the Tuebingen data with 610.352 Hz. The sensor arrangement in both systems is designed to fit the shape of the pregnant women’s abdomen. Before each fetal measurement, an ultrasound is performed to determine fetal head position. Afterward, the mothers are seated on the fMEG device in the shielded room. Prior to the recording localization coils are placed at the left side, the right side and the spine of the mother as well as at the position on the abdomen where the fetal head is located. These coils indicate the maternal as well as the fetal position relative to the sensor array which are used in later data analysis.

### Participants from tuebingen study (first part)

Twenty-five healthy pregnant women with uncomplicated pregnancies and normally developing fetuses (*M*_age_ = 34.59 weeks, SD ± 2.35 weeks) between the gestational age of 30 and 38 weeks and 28 healthy newborns and infants without complications during pregnancy and birth between the age of 14 and 67 days (*M*_age_ = 37.18 days SD ± 15.52) participated in the study. The local ethical committee approved the study and a written consent was obtained.

### Protocol used in tuebingen study (first part)

**Figure 1:**
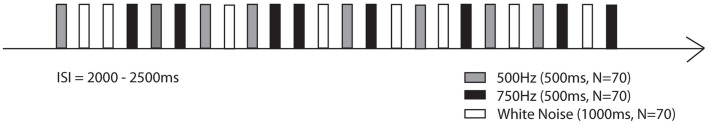
**Stimulation paradigm of the first part of the study (Tuebingen)**.

For neonatal/infant measurements, a cradle was attached to the fMEG devise and neonates/infants were lying on their right side with their right hemisphere over the sensor array. Auditory stimulation was generated outside the shielded room and transmitted through flexible, air-filled tubes into the shielded room. Stimulation was presented with a sound pressure level of 65 dB to the left ear using earphones especially developed for neonatal measurements (bio-logic, USA). Stimulation consisted of the random presentation of a 500 Hz tone (*n* = 70), a 750 Hz tone (*n* = 70), and WN (*n* = 70). The stimulus duration was 0.5 s for the 500 Hz tone and the 750 Hz tone and 1 s for the “WN” (80–1000 Hz) segments. The inter-stimulus interval (ISI) between stimulus presentations varied randomly between 2 and 2.5 s. The pure stimulation duration was 8 min (Figure 1).

All neonates/infants were measured while sleeping or lying quietly. During the whole measurement, one parent was inside the shielded room with the baby.

For the fetal measurements, the same paradigm was applied. Stimulation was presented using an air-filled balloon, which was placed directly on the maternal abdomen. Stimulation intensity was 95 dB, which is attenuated by maternal tissue and amniotic fluid and which the fetus may perceive at a level of approximately 65–70 dB (Querleu et al., 1988).

### Participants from little rock study (second part)

Twelve healthy pregnant women with uncomplicated pregnancies and normally developing fetuses (*M*_age_ = 25.7 weeks GA, SD ± 2.4) and six healthy newborns and infants without complications during pregnancy and birth (*M*_age_ = 23 days and SD ± 6.2) participated in the study. The local ethical committee approved the study and a written consent was obtained.

### Protocol used in little rock study (second part)

**Figure 2:**
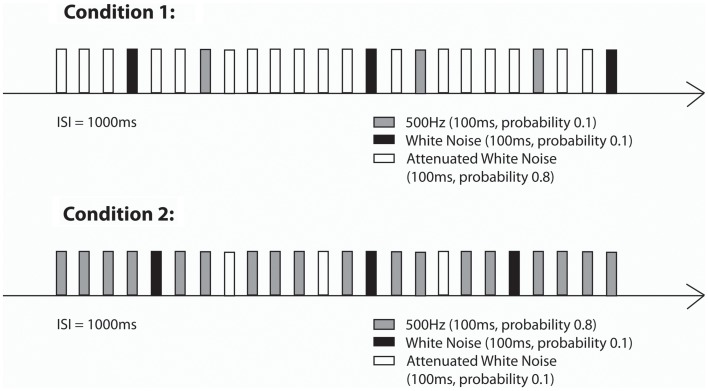
**Stimulation paradigms (Condition 1 and Condition 2) of the second part of the study (Little Rock)**.

Auditory stimulation was generated outside the shielded room and the stimuli were delivered to the inside of the shielded room through Tygon tubing that ended with an air-filled bag. Similar to the first paradigm, for the neonatal measurements a cradle was attached to the fMEG device and neonates/infants were lying on their side with one hemisphere over the sensor array. Stimulation was presented with a sound pressure level of 65 dB at the end of the acoustic emitter, which was attached to the ceiling at a distance of approximately 75 cm from the newborn’s head. The stimulation paradigm consisted of two oddball conditions in which a 500 Hz tone, attenuated WN and WN were either standards (probability of 0.8, presented for 560 times) or deviant sounds (each type with a probability of 0.1, presented for 70 times) (see Figure 2). Total number of presented stimuli was 700. The stimulus duration was 0.1 s. The ISI was 1 s. The total stimulation duration was about 17 min per condition. All neonates were measured while sleeping or lying quietly. During the measurement, one parent was inside the shielded room with the baby.

For fetal measurements, the same paradigm was used. Sound intensity measured at the end of the tube in the air was 115 dB for the 500 Hz tones as well as for the WN segments. For the attenuated WN segments the sound intensity at the end of the tube was 95 dB.

### Data analysis

All fMEG recordings were filtered offline using a bandpass filter between 1 and 10 Hz for fetal recordings and between 1 and 15 Hz for neonatal recordings using Butterworth filter with zero phase distortion. Interfering maternal and fetal cardiac signals were attenuated using an orthogonal signal space projection technique (Vrba et al., 2004; McCubbin et al., 2006) or a Hilbert transform method (Wilson et al., 2008). Data was cut in segments according to the test stimulus; the length of the segments was 200 ms before the stimulus (baseline) and 800 ms after the stimulus. All segments including artifacts due to maternal or fetal movement or muscle contractions higher than 2 pT were excluded from further analysis. Evoked responses were determined by visual inspection and quantified by calculating the root-mean squares (RMS) of the five channels with the highest amplitudes. These RMS were used for statistical analysis.

### Statistical analysis

For the Tuebingen Study, the RMSs of all conditions were tested for normal distribution. For normally distributed data, a repeated-measures ANOVA with “tones” as within-subject factor was used to test for main effects and paired *t*-tests were used for *post hoc* comparisons. For non-normally distributed data, the non-parametric Friedman test was used to test for main effects. Significance levels were adjusted for multiple comparisons using Bonferroni correction.

Due to the explorative nature and the small sample size of the Little Rock study, the statistical analysis of this part had to be limited to descriptive indices and non-parametric comparative tests.

## Results

### Results from tuebingen study (first part)

#### Neonatal/infant recordings

Fifteen neonates/infants were excluded from further analysis because either no measurement was possible due to movement/crying or because measurements had to be aborted due to agitation of the neonate/infant. Statistical analysis has been done on the remaining 13 datasets (*M*_age_ = 34.08 days, SD ± 15.18).

**Figure 3:**
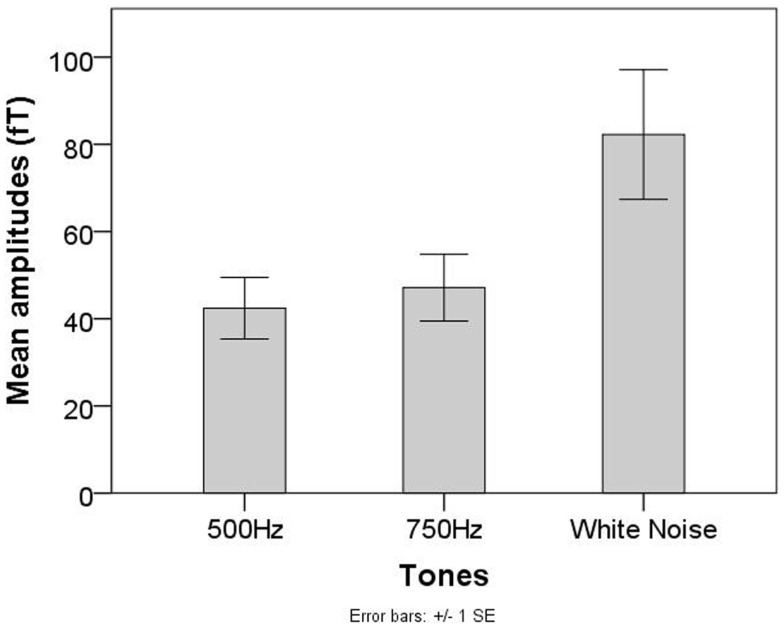
**Mean AER amplitudes for the 500 Hz, 750 Hz, and WN stimuli in the group of neonates/infants of the Tuebingen study**.

A significant main effect of tones was observed (*F* = 11.45, *p* < 0.05). *Post hoc* analysis revealed significant differences between the 500 Hz tone and WN (*T*_12_ = −3.82, *p* < 0.0167) and the 750 Hz tone and WN (*T*_12_ = −3.22, *p* < 0.0167). No significant differences between the 500 Hz tone and the 750 Hz tone were observed (*T*_12_ = −1.05, *p* = 0.317, see Figure 3).

#### Fetal recordings

Eleven fetuses were excluded from further analysis because they did not show visible evoked responses for all three conditions. All further analysis is based on the remaining 14 fetuses (*M*_age_ = 34.57, SD ≤ 2.34).

**Figure 4:**
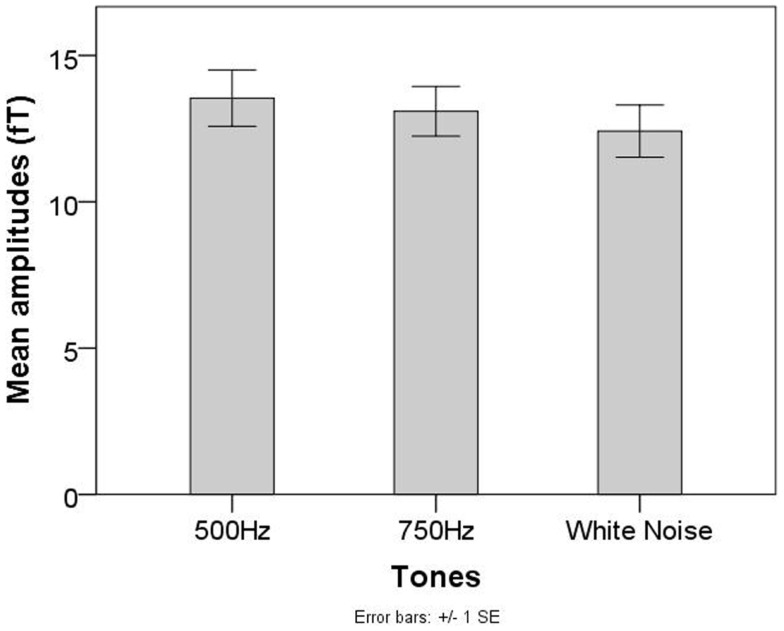
**Mean AER amplitudes for the 500 Hz, 750 Hz, and WN stimuli in the group of fetuses of the Tuebingen study**.

No significant main effect of tones was found for fetal recordings (χ^2^ = 2.71, *p* = 0.26, see Figure 4).

### Results from little rock study (second part)

#### Neonatal/infant recordings

One out of six neonatal/infant recordings was excluded from the analysis because it was stopped within the first 5 min of the measurement due to newborn crying. The RMS values of the amplitudes of the AERs for the neonates are shown in Table 1.

**Table 1:** **RMS values of the neonatal AERs for both oddball conditions applied in the second stimulation paradigm**.

Subject	First oddball condition		Second oddball condition	
RMS	WN (fT)	500 Hz (fT)	WN (fT)	WN attenuated (fT)
WN01	30.70	14.99	17.90	11.49
WN02	33.50	27.30	30.70	26.40
WN03	42.20	29.20	67.10	29.32
WN04	38.00	31.10	42.30	35.68
WN05	44.00	40.70	45.00	36.20

Mean RMS value for the WN segments in the first oddball condition (*M* = 37.7, SD ± 5.6) was higher than the mean of the RMS values for the 500 Hz tones (*M* = 28.7, SD ± 9.2). The same was true for the second condition between the amplitudes of the WN segments (*M* = 40.6, SD ± 18.3) and those of the attenuated WN segments (*M* = 27.8, SD ± 0.04). Wilcoxon signed ranked tests showed significant differences (*Z* = 3.7, *p* < 0.05) between the median amplitudes of the AERs to the WN and those to the attenuated WN segments as well as between the amplitudes of the cortical responses to the WN and those to the 500 Hz tones of the second condition (*Z* = 2.02, *p* < 0.05).

#### Fetal recordings

Two out of 12 fetal recordings were excluded from further analysis based on maternal movements and large maternal and fetal heart residuals. AERs on the deviants were detected in 8 out of 10 fetuses in the first oddball condition and in 5 out of 10 fetuses in the second one. Amplitudes of the AERs elicited by infrequent WN segments were not significantly different than those elicited by the infrequent 500 Hz tones (*Z* = −0.70, *p* = 0.48). The difference between RMS values of AERs elicited by infrequent WN segments and those elicited by infrequent attenuated WN segments showed a tendency toward significance (*Z* = −1.8, *p* = 0.08).

## Discussion

The current study aimed to use a simplified paradigm to replicate the increased neonatal reaction to WN stimuli which was already found by Kushnerenko et al. (2007). Moreover, WN segments were used for the first time in fetal recordings to investigate if the human brain is able to detect changes in the spectral width of auditory stimulation during fetal period. An oddball paradigm including changes in volume and spectral width was used to further investigate fetal change detection.

Results of the first paradigm showed, that even when using a simple paradigm wherein pure tones and WN segments were presented in random order with the same probability, infants showed significantly higher reactions to the WN segments than to the pure tones. This replicates prior results showing that infants are sensitive to the effective amount of stimulation with higher reactions to high-energy stimulation (in this case high spectral width) and lower reactions to low-energy stimulation (Kushnerenko et al., 2007). Also earlier behavioral studies support these findings (Lewkowicz and Turkewitz, 1980). Even though our results are in line with other studies, it has to be taken into account that the duration of the WN segments was longer than that of pure tones in the current study. Therefore, the higher reaction to the WN segments might partly be explained by the change in duration rather than in spectral width. However, when studying change detection using a multiple deviant paradigm, Sambeth et al. (2009) found no significant differences between amplitudes of the reaction to standards and duration deviants using magnetoencephalography. This indicates that a change in tone duration plays only a minor role concerning the increment of AER amplitudes. Our results from the second paradigm further support this finding. Neonatal/infant cortical responses to WN segments showed significantly higher amplitudes when compared to pure tones and attenuated WN segments of the same duration. Nevertheless, additional data is needed to thoroughly address this question.

Different from neonates and infants, in both fetal paradigms stimuli consisting of multiple frequencies did not elicit AERs with higher amplitudes than pure tones. Special requirements for fetal measurements might be one important factor concerning the evaluated group differences between neonates and fetuses. While neonates can be stimulated directly at the ear with sounds transmitted through the air, fetal stimulation takes place outside the maternal abdomen. Since the fetus is surrounded by amniotic fluid, the characteristics of sound transmission are different than sound transmission in air. Different studies have been performed to evaluate sound attenuation in fetal measurements [Hepper and Shahidullah (1994) and references therein]. Using a hydrophone near the fetal head at the beginning of labor and after amniotomy, Querleu et al. (1988) recorded an attenuation of 2 dB at 250 Hz, 14 dB at 500 Hz, 20 dB at 1000 Hz, and 26 dB at 2000 Hz. Generally, higher frequencies are more attenuated than lower frequencies, which in some works are even reported to be slightly enhanced in volume (Richards et al., 1992). Since the WN segments in the current study were composed of frequencies in the range between 50 and 1000 Hz, which were all presented with the same sound pressure level, we assume that the higher frequencies were attenuated much more than the lower frequencies before they reached the fetal ear. Compared to neonates/infants who heard the full spectral width of WN segments, fetuses might have perceived only parts of the frequency range, narrowed down to lower and middle frequencies. In this case, due to a narrowed perceived spectral width, also the total energy of the stimulation would have been weakened, leading to AER amplitudes comparable with those of pure tones. Additionally to the differences in sound transmission between fetuses and neonates, the background noise differs between those groups. While neonates are stimulated in a quiet environment, fetuses are surrounded by background noises caused by the maternal heart beat, bowel movements or the maternal voice. Different studies reported background noise intensities of 72 dB measured during labor in the uterus (Bench, 1968), 85 dB measured before labor, and even 95 dB after the R-wave of the maternal heart (Walker et al., 1971). Since these background noises are of low frequencies, we believe that they might have superimposed and masked the lower frequency ranges of WN segments, leaving the higher frequencies nearly unaffected. Since the low frequencies are less attenuated but covered by the background noise, the perceived frequency range is additionally narrowed down. Even though learning studies in fetuses (Partanen et al., 2013a,b) clearly showed that fetuses are able to perceive and learn melodies and speech sounds, these studies did not focus on the effective amount of stimulation. Even if a high range of stimuli can be perceived when presented separately, no clear conclusion can be drawn concerning the frequencies perceived by the fetus when a broad range of frequencies are presented at the same time with the same intensity. Further research is needed to entirely solve this question.

Taken together, we assume that fetuses probably did not perceive the full frequency range of the WN stimulus, which weakened stimulus energy and made it less distinguishable from the pure tone stimuli. The results of the second study suggest that the neonates are sensitive to both spectral and volume changes, whereas the fetuses are rather sensitive to loudness change.

It has to be stressed that the two studies were performed with two separate systems for fMEG recordings and the results were comparable, providing further support for the fMEG as a reliable method to assess fetal brain development.

In summary, the current study showed that newborns are able to differentiate between pure tones and WN segments even in simple paradigms, where tones are randomly presented with the same probability. This enables shorter measurement times, which is an important point in neonatal measurements since movement artifacts are highly probable especially in long measurements. To thoroughly evaluate fetal ability to distinguish between high- and low-energy stimulation, stimulation methods need to be developed which are not attenuated by the maternal abdomen and amniotic fluid and therefore ensure that the fetus receives the complete spectrum of the WN.

## Authors Contribution

Jana Muenssinger was involved in the development of the design, data acquisition and analysis, statistical analysis, and wrote the manuscript. Tamara Matuz was involved in the development of the design, data acquisition and analysis, statistical analysis, and writing of the manuscript. Franziska Schleger was involved in data acquisition and analysis and revised the manuscript. Rossitza Draganova was involved in the data analysis and revised the manuscript. Magdalene Weiss was involved in the recruitment of subjects, data acquisition, and revised the manuscript. Isabelle Kiefer-Schmidt and Annette Wacker-Gussmann were involved in the recruitment of subjects and revised the manuscript. Rathinaswamy B. Govindan was involved in the development of the paradigm, data analysis, and revised the manuscript. Curtis L. Lowery was involved in the conceptual design of the study and revised the manuscript. Hari Eswaran was involved in designing the study, analyzing and interpreting results, and revised the manuscript. Hubert Preissl was involved in the development of the design, data analysis and revised the manuscript.

## Conflict of Interest Statement

The authors declare that the research was conducted in the absence of any commercial or financial relationships that could be construed as a potential conflict of interest.
